# Worthy of Sympathy and Support

**Published:** 1887-02-12

**Authors:** 


### Worthy of Sympathy and Support.

C. J. F. writes:—"There is a girl (the younger sister of a servant of mine) in whom I am somewhat interested. Her parents are very poor, but respectable, hardworking people; the girl in question suffers very much from her sight; sometimes it is so bad that she can hardly see her way about. She also suffers from ulcers: one now upon her leg is several inches long. She is a very good girl, and it is exceedingly sad to see her so afflicted. She is but nine years old, and if she could obtain good food and careful treatment in a hospital for a time, I daresay she could be made quite another child. It occurred to me that perhaps you might be so kind as to use your influence, or to offer me any suggestion on behalf of this poor child. Her friends, who find existence an exceedingly hard struggle, would be deeply grateful. They have a young family of nine."

